# A Narrative Review of the Efficacy and Safety of Oral Ketamine in Pediatric Sedation: A Critical Analysis of Current Evidence

**DOI:** 10.7759/cureus.67550

**Published:** 2024-08-22

**Authors:** Lakshmi Naga Sai Sivani Dasari, Sanjot Ninave

**Affiliations:** 1 Anesthesia and Critical Care, Jawaharlal Nehru Medical College, Datta Meghe Institute of Higher Education and Research, Wardha, IND

**Keywords:** non-invasive sedation, sedative agents, safety, efficacy, pediatric sedation, oral ketamine

## Abstract

Sedation in pediatric patients presents unique challenges due to their developmental and physiological differences compared to adults. Oral ketamine, a dissociative anesthetic, has emerged as a promising alternative to traditional sedatives, offering a non-invasive method for achieving sedation in children. This comprehensive review evaluates the efficacy and safety of oral ketamine for pediatric sedation, consolidating evidence from recent studies and clinical trials. The review details the pharmacological properties of oral ketamine, including its mechanism of action and its role in achieving effective sedation. It examines dosing guidelines, clinical applications, and the outcomes of sedation procedures utilizing oral ketamine. Additionally, the review addresses the safety profile of oral ketamine, including standard and serious adverse effects, and provides recommendations for monitoring and managing potential risks. Comparative analyses with other sedation methods highlight the advantages and limitations of oral ketamine, including its effectiveness and ease of administration compared to intravenous (IV) and inhaled sedatives. The review also identifies gaps in the current literature and suggests areas for future research, including long-term safety and potential developmental impacts. In conclusion, oral ketamine represents a valuable option for pediatric sedation, offering a balance of efficacy and ease of use. This review aims to guide clinicians in making informed decisions regarding the use of oral ketamine, contributing to safer and more effective sedation practices in pediatric care.

## Introduction and background

Sedation in pediatric patients is a crucial aspect of medical care, enabling the safe and effective performance of procedures while ensuring patient comfort. Given the unique physiological and developmental characteristics of children, achieving the right level of sedation is both an art and a science [1]. Traditional methods, such as intravenous (IV) sedatives and inhaled anesthetics, are commonly used but often require specialized equipment and skilled personnel. These methods can be challenging to administer, especially in settings where resources are limited [2]. As a result, there is increasing interest in alternative sedative agents that can provide effective sedation with greater ease of administration. Oral ketamine has emerged as a potential option due to its distinct pharmacological properties and its ability to be administered non-invasively [3].

The purpose of this comprehensive review is to critically evaluate the efficacy and safety of oral ketamine for pediatric sedation. Ketamine, a dissociative anesthetic with a unique mechanism of action, has been explored for its potential benefits in this context. This review seeks to consolidate existing research to provide a clear understanding of how oral ketamine performs as a sedative in pediatric settings. By assessing available evidence, the review aims to inform clinicians about the practical applications of oral ketamine, its effectiveness in achieving adequate sedation, and its safety profile for children [4].

This review is specifically focused on the use of oral ketamine for pediatric sedation, excluding other forms of ketamine administration, such as IV or intranasal routes. The review will cover various aspects, including dosing, efficacy, safety, and clinical applications of oral ketamine in children. However, there are limitations to consider. The availability of high-quality, recent studies on this topic may be limited, and variations in study design and methodologies could influence the findings. The results may not be universally applicable due to differences in protocols and practices across different healthcare settings. Despite these limitations, this review aims to provide a balanced and thorough examination of the role of oral ketamine in pediatric sedation.

## Review

Mechanism of action

**Figure 1:**
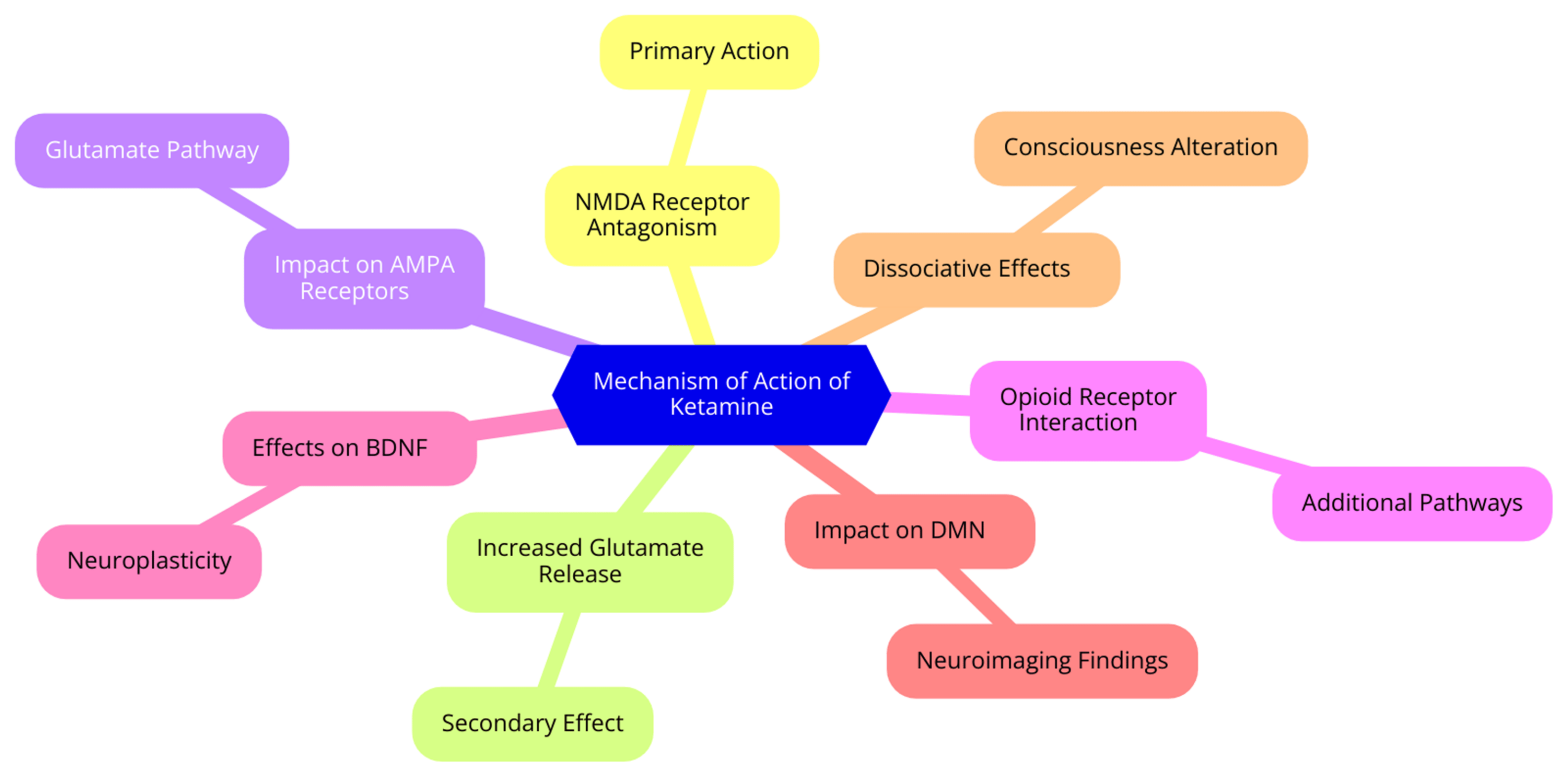
The mechanism of action of ketamine Image credit: Dr. Lakshmi Naga Sai Sivani Dasari NMDA: N-methyl-D-aspartate; AMPA: α-amino-3-hydroxy-5-methyl-4-isoxazolepropionic acid; BDNF: brain-derived neurotrophic factor; DMN: default mode network

Ketamine's mechanism of action is multifaceted, primarily as an antagonist of N-methyl-D-aspartate (NMDA) receptors in the central nervous system. These receptors are critical for pain perception and modulation, and by blocking them, ketamine effectively inhibits the transmission of pain signals. This blockade is a key reason for its potent analgesic effects, making it particularly useful in surgical settings and pain management for pediatric patients [5]. In addition to NMDA receptor antagonism, ketamine influences various neurotransmitter systems throughout the brain. One significant interaction occurs with opioid receptors, where ketamine binds to mu and delta receptors, further enhancing its analgesic properties [6]. Moreover, ketamine has been shown to enhance descending inhibitory serotonergic pathways, which not only contribute to its analgesic effects but may also provide antidepressant-like benefits. This interaction with the serotonergic system highlights ketamine's potential beyond traditional anesthetic applications [6]. Ketamine also interacts with muscarinic acetylcholine receptors, which may contribute to its dissociative and psychotomimetic effects. Additionally, it affects voltage-sensitive calcium channels, a mechanism that may play a role in its anesthetic and analgesic properties [7]. Another interesting aspect of ketamine's action is its inhibition of nitric oxide synthase in the brain, which could be linked to its potential antidepressant effects. Furthermore, ketamine modulates hyperpolarization-activated cyclic nucleotide-gated (HCN1) channels, influencing neuronal excitability and contributing to its hypnotic actions [7]. The mechanism of action of ketamine is shown in Figure 1.

Efficacy of oral ketamine

Oral ketamine is increasingly recognized for its efficacy and safety in pediatric sedation. This review explores its clinical applications, dosing recommendations, and effectiveness compared to other sedatives [8]. Oral ketamine is utilized in various clinical scenarios, particularly in pediatric settings. It is effective for preoperative sedation in children undergoing day-case surgeries, facilitating easier separation from parents and acceptance of anesthetic masks [4]. Ketamine is also employed during painful procedures such as IV cannulation, where it significantly reduces pain and distress in children, making procedures easier to conduct. Its use is also noted in emergency departments for short, painful procedures, where it is a potent sedative and analgesic agent [9]. For premedication in children, doses of 5 mg/kg to 10 mg/kg are commonly used. A study found that 10 mg/kg resulted in better sedation and anxiety relief than lower doses. Initial doses for procedural sedation typically range from 1 to 1.5 mg/kg, with the option for incremental doses of 0.25-0.5 mg/kg every 10 minutes if needed. The maximum dose may reach 4.5 mg/kg, which is rarely required. Oral ketamine can be administered via multiple routes, including oral (commonly used for premedication and pain management), IV (preferred for rapid onset in procedural sedation), and intramuscular (IM) (used when IV access is not feasible) [10]. Oral ketamine has demonstrated high success rates in achieving adequate sedation and anxiolysis. Studies involving premedication showed satisfactory sedation in 84% of children receiving 10 mg/kg, with similar rates noted during painful procedures. When compared to other sedatives like midazolam, oral ketamine has shown advantages in certain contexts. For instance, a combination of ketamine and midazolam has been reported to yield less sedation failure and faster recovery times. Additionally, oral ketamine has been associated with fewer side effects, making it a favorable option for pediatric sedation [11].

Safety profile

The safety profile of oral ketamine is a crucial consideration in its use for pediatric sedation. Understanding the potential adverse effects, monitoring requirements, and long-term safety implications helps ensure its safe application in clinical settings [12]. Oral ketamine is generally well-tolerated in pediatric patients, but some common side effects may include drowsiness, nausea and vomiting, and increased salivation. Serious side effects are rare but can include respiratory depression and cardiovascular effects. One of the notable concerns with ketamine use, particularly in higher doses, is the potential for psychological effects, including hallucinations and dissociative symptoms [13]. Effective monitoring is essential to ensure patient safety during sedation with oral ketamine. Recommended guidelines include continuous monitoring of vital signs, regular assessments of the child's level of consciousness, and post-sedation recovery observation in a controlled environment until the child is fully alert and stable. Several management strategies can be employed in the event of adverse effects, such as administering antiemetic medications for nausea and vomiting, providing a calm environment and reassurance for hallucinations, and immediate intervention for respiratory or cardiovascular issues [14]. While short-term use of ketamine in pediatric sedation is safe, the long-term effects on development and cognitive function are still a subject of ongoing research. Current findings suggest that there is limited evidence indicating that ketamine, when used appropriately, does not adversely affect cognitive development in children. However, some studies have suggested potential behavioral changes following repeated exposure, necessitating careful consideration of the frequency and context of use [12]. The risk of abuse and dependency in pediatric populations is lower compared to adults but remains a concern. Ketamine is a Schedule III controlled substance in many jurisdictions, and its dissociative effects may lead to misuse. Long-term use, particularly in non-medical settings, can lead to psychological dependence. Clinicians should be vigilant in assessing the necessity of continued use and consider alternative sedation options when appropriate [15].

Comparison with other sedatives

Oral Ketamine Versus Other Oral Sedatives

When comparing oral ketamine to other oral sedatives, such as midazolam, notable differences in efficacy and safety emerge. A study evaluating oral ketamine (5 mg/kg) versus oral midazolam (0.7 mg/kg) for sedation during laceration repair found that both agents provided adequate sedation. However, a significant percentage of children receiving ketamine (32%) required additional IV sedation, compared to only 6% of those treated with midazolam. This suggests that while oral ketamine can be effective, it may have a higher failure rate as a standalone agent compared to midazolam [11]. Interestingly, research indicates that combining oral midazolam with ketamine can enhance sedation outcomes. In one study, this combination resulted in better sedation levels than midazolam alone, highlighting the potential for ketamine to be used effectively in conjunction with other sedatives. Regarding safety, both oral ketamine and midazolam exhibited low incidences of adverse effects, such as nausea and vomiting, with no significant differences in side effects between the two medications. This suggests that oral ketamine is a safe option, although its efficacy may be improved through combination therapy [16].

Oral Ketamine Versus Parenteral Sedatives

The comparison between oral ketamine and parenteral sedatives reveals significant differences in efficacy, onset, and duration of action. Parenteral administration of ketamine provides much higher bioavailability, approximately 93%, compared to only about 17% for oral dosing due to extensive first-pass metabolism. This difference in pharmacokinetics significantly impacts the medication’s efficacy [17]. For instance, studies indicate that oral ketamine at a dose of 10 mg/kg can achieve satisfactory sedation within approximately 30 minutes, whereas parenteral ketamine typically acts within 1-2 minutes of IV administration [4]. The duration of sedation also varies between the two routes of administration. Oral ketamine may have a longer duration of action due to its extended absorption phase, making it suitable for procedures requiring prolonged sedation [18]. However, it is important to note that parenteral ketamine is associated with higher incidences of psychomimetic emergence reactions, such as hallucinations and agitation, which appear to be less frequent with oral dosing. In summary, while oral and parenteral ketamine share similar mechanisms of action, the differences in pharmacokinetics result in variations in efficacy, onset, and duration, making oral ketamine a valuable option in pediatric sedation, particularly when the rapid onset is not critical [3].

Evidence from recent studies

Review of Recent Literature

Recent studies have explored the efficacy and safety of oral ketamine in pediatric sedation, particularly in comparison to other sedatives like midazolam, and its role in various clinical settings [19]. One notable study assessed oral ketamine at a dose of 5 mg/kg against oral midazolam at 0.7 mg/kg for laceration repair in children aged one to 10 years. The findings revealed that ketamine had a higher sedation failure rate, with 32% of children requiring additional IV sedation compared to only 6% for midazolam. Although both agents provided similar parent-assessed pain levels, this suggests that while ketamine can be effective for sedation, it may not be as reliable as midazolam for certain procedures [11]. In exploring combination therapy, some research has indicated that using low doses of both oral ketamine and midazolam can enhance sedation reliability and minimize adverse effects. This potential synergistic effect may improve outcomes in pediatric sedation, making it a promising avenue for further investigation [20]. Additionally, studies have highlighted ketamine's effectiveness in pain management during procedural sedation. In one study involving 160 children, those receiving oral ketamine reported significantly lower pain scores compared to a placebo group, with adverse effects being generally mild and transient, reinforcing ketamine's safety profile [20]. The optimal dosing for oral ketamine appears to be in the range of 6-10 mg/kg, as research indicates that higher doses lead to better sedation outcomes without significantly increasing adverse effects. However, careful monitoring is essential to manage potential side effects such as nausea and vomiting. These findings underscore the importance of individualized dosing and the need for healthcare providers to weigh the risks and benefits when considering oral ketamine for pediatric sedation [3].

Analysis of Findings

Recent studies highlight several key points regarding the use of oral ketamine in pediatric sedation. First, while oral ketamine can provide adequate sedation, its reliability is sometimes questioned, particularly when compared to midazolam. The higher failure rate in achieving satisfactory sedation indicates that ketamine may not be suitable as a standalone agent for all procedures, especially those requiring higher levels of sedation [12]. Moreover, combining ketamine with midazolam appears to enhance sedation efficacy and reduce the likelihood of sedation failure, suggesting that using multiple agents may be beneficial in pediatric settings. This approach allows for more effective management of anxiety and pain during procedures, ultimately leading to improved patient experiences [21]. Safety is another critical aspect highlighted by the studies. Oral ketamine is associated with a favorable safety profile, with most adverse effects being mild and transient. Serious complications are rare, making it a viable option for procedural sedation in children. This safety profile is particularly important in pediatric populations, where the risk of adverse effects can be a significant concern [22]. Finally, the studies suggest that an optimal dosing range of 6-10 mg/kg is effective for sedation, with higher doses potentially offering better outcomes. However, the risk of side effects necessitates careful consideration of individual patient factors. In conclusion, while oral ketamine shows promise in pediatric sedation, particularly when combined with other agents, further research is needed to clarify its role and optimize its use in clinical practice [23].

Clinical guidelines and recommendations

Current Guidelines

The use of oral ketamine for pediatric sedation is not extensively covered in major clinical guidelines, but important insights can be drawn from the available literature. The American Society of Regional Anesthesia and Pain Medicine (ASRA) published consensus guidelines in 2018 that briefly address oral ketamine. They concluded that there is low-level evidence supporting the use of oral ketamine for chronic pain management at doses around 150 mg/day or 0.5 mg/kg every six hours. For acute pain management, the evidence is limited, but it suggests that oral ketamine may provide short-term benefits [24]. In contrast, guidelines from the Royal Children's Hospital in Melbourne, Australia, recommend oral ketamine as a safe and effective option for procedural sedation in pediatric patients, particularly in emergency department settings. These guidelines suggest an initial dosing regimen of 1-1.5 mg/kg, possibly administering subsequent incremental doses of 0.25-0.5 mg/kg every ten minutes as needed, up to a maximum of 4.5 mg/kg. This framework provides a practical approach for clinicians considering oral ketamine for sedation in children [25].

Best Practices

Based on the available evidence, several best practices can be recommended for clinicians administering oral ketamine for pediatric sedation. First, precise dosing is essential. An initial dose of 1-1.5 mg/kg is generally recommended, with the possibility of incremental doses of 0.25-0.5 mg/kg administered every ten minutes, not exceeding a total of 4.5 mg/kg. Research suggests that higher doses, such as 5-10 mg/kg, may enhance sedation and anxiolysis, with the 10 mg/kg dose showing superior outcomes compared to lower doses [4]. Monitoring is a critical aspect of ensuring safe sedation practices. Clinicians should continuously observe vital signs throughout the procedure, including heart rate, respiratory rate, and blood pressure. Employing a validated sedation scale, such as the 5-point sedation score, can help accurately assess the child's level of sedation. Additionally, monitoring for potential adverse effects, such as nausea, vomiting, or emergency reactions, is important, although these side effects are generally rare with oral administration [26]. Implementing comprehensive safety protocols is vital for minimizing risks associated with oral ketamine use. Clinicians should ensure that resuscitation equipment is readily available to address adverse reactions promptly. Consulting with senior staff or anesthesia colleagues is advisable if the child has specific risk factors, such as severe asthma, head injury, or cardiovascular issues. Furthermore, the child should be closely observed and supervised for at least two hours post-procedure until they can walk and return to their baseline level of consciousness [24].

Future directions

The field of pediatric sedation presents numerous research opportunities, with several critical gaps that need addressing. A major gap is the lack of comprehensive evidence from well-designed, adequately powered randomized controlled trials (RCTs) evaluating the efficacy and safety of various sedative agents and their combinations in pediatric populations, particularly among critically ill children [27]. Additionally, there is a pressing need to assess whether protocolized sedation improves clinical outcomes, including both short-term metrics like ventilator-free days and long-term effects such as neurodevelopmental outcomes and the risk of post-traumatic stress disorder (PTSD) in pediatric patients. Optimal dosing strategies for sedatives in diverse pediatric populations, especially those with underlying health conditions, also require further investigation. Furthermore, special populations, such as children with autism or intellectual disabilities, are often underrepresented in sedation research, highlighting the need for targeted studies to develop safe and effective protocols tailored to their unique needs [28]. Several improvements can be made to enhance the safety and efficacy of pediatric sedation. Future research should focus on robust study designs, including multi-center collaborations, to achieve adequate sample sizes and improve the generalizability of findings. Validated sedation scales designed for pediatric populations can help assess sedation levels accurately. Additionally, improved training for healthcare providers in sedation practices is essential to ensure they can effectively implement new protocols and monitor for adverse effects. Investigating novel sedative agents, such as dexmedetomidine, may offer alternatives with better safety profiles and efficacy, warranting research into their use in various sedation contexts, including outpatient settings [29]. Emerging trends in pediatric sedation are shaping the future of this field. There is an increasing emphasis on moderate sedation techniques, which aim to balance between general anesthesia and awake procedures to enhance patient comfort while minimizing the risks associated with deeper sedation. Advances in technology, particularly in monitoring systems and sedation delivery methods, enable more precise control over sedation levels and improve patient monitoring during procedures. This progress significantly enhances the safety and efficacy of pediatric sedation practices. Additionally, a growing focus on conscious sedation in pediatric dentistry reflects a broader trend toward evidence-based practices for managing pediatric patients undergoing dental procedures. These developments highlight the importance of ongoing research and innovation to ensure the safety and effectiveness of sedation practices for this vulnerable population [30].

## Conclusions

In conclusion, oral ketamine presents a promising alternative for pediatric sedation, offering a non-invasive and effective option for managing procedural discomfort and anxiety in children. The current evidence suggests that oral ketamine can provide adequate sedation with a favorable safety profile. However, it is essential to consider the variability in individual responses and potential side effects. While oral ketamine offers several advantages, such as ease of administration and reduced need for specialized equipment, further research is necessary to fully understand its long-term effects and optimize dosing strategies. Clinicians must weigh the benefits against potential risks and adhere to established safety protocols to ensure the best outcomes for pediatric patients. As pediatric sedation continues to evolve, ongoing studies and clinical experience will further refine our understanding of oral ketamine's role, ultimately contributing to improved sedation practices and enhanced patient care.
